# Enantioselective Synthesis of 8-Hydroxyquinoline Derivative, Q134 as a Hypoxic Adaptation Inducing Agent

**DOI:** 10.3390/molecules24234269

**Published:** 2019-11-23

**Authors:** László Hackler, Márió Gyuris, Orsolya Huzián, Róbert Alföldi, Gábor J. Szebeni, Ramóna Madácsi, Levente Knapp, Iván Kanizsai, László G. Puskás

**Affiliations:** 1 Avidin Ltd., 6726 Szeged, Hungarym.gyuris@avidinbiotech.com (M.G.); g.szebeni@avidinbiotech.com (G.J.S.); r.madacsi@avicorbiotech.com (R.M.); i.kanizsai@avidinbiotech.com (I.K.); 2 Avicor Ltd., 6726 Szeged, Hungary; o.huzian@avicorbiotech.com (O.H.); r.alfoldi@avidinbiotech.com (R.A.); l.knapp@avicorbiotech.com (L.K.); 3 Aperus Pharma Co. Ltd., 6726 Szeged, Hungary

**Keywords:** 8-hydroxyquinoline, Alzheimer’s disease, Betti reaction, enantioselective synthesis, cytoprotection, neurodegeneration, mitochondrial membrane potential, HIF1A

## Abstract

Hypoxia is a common feature of neurodegenerative diseases, including Alzheimer’s disease that may be responsible for disease pathogenesis and progression. Therefore, the hypoxia-inducible factor (HIF)1 system, responsible for hypoxic adaptation, is a potential therapeutic target to combat these diseases by activators of cytoprotective protein induction. We have selected a candidate molecule from our cytoprotective hydroxyquinoline library and developed a novel enantioselective synthesis for the production of its enantiomers. The use of quinidine or quinine as a catalyst enabled the preparation of enantiomer-pure products. We have utilized in vitro assays to evaluate cytoprotective activity, a fluorescence-activated cell sorting (FACS) based assay measuring mitochondrial membrane potential changes, and gene and protein expression analysis. Our data showed that the enantiomers of Q134 showed potent and similar activity in all tested assays. We have concluded that the enantiomers exert their cytoprotective activity via the HIF1 system through HIF1A protein stabilization.

## 1. Introduction

Alzheimer’s disease (AD) is an irreversible, progressive neurodegenerative disease of the brain which leads to loss of memory and cognitive functions. The pathological hallmarks of the disease are amyloid beta deposition, neurofibrillary tangles, neuroinflammation, and neuronal loss in the patient’s brain [1]. Two forms of AD are defined generally: a familial early-onset form linked to gene mutations in amyloid β precursor protein and presenilin and a sporadic late-onset form. More evidence suggests that environmental risk factors play an important role in the onset and development of sporadic AD which constitute the majority of AD cases [2,3].

Hypoxia is a common etiology in neurodegenerative diseases, including AD [4]. Occurrence and susceptibility to AD is higher in individuals suffering from cerebral ischemia and stroke in which hypoxic conditions occur. Hypoxia may be responsible for promoting the pathogenesis and progression of AD through several factors: increasing the processing and inhibiting the clearance of amyloid beta, promoting tau hyperphosphorylation and facilitation of neuronal death [5,6].

Reduction of blood supply leading to hypoxic condition is known to activate cellular responses mainly controlled by hypoxia-inducible factors (HIFs). Hypoxia-inducible factors are heterodimeric transcription factors consisting of an α- and a β-subunit belonging to the basic helix-loop-helix Per-Arnt-Sim (bHLH)-PAS protein superfamily. Hypoxia-inducible factor 1 alpha (HIF1A) protein level is regulated by protein degradation and stabilization under normoxic or hypoxic conditions. HIF1A is stabilized under low O_2_ levels through the inhibition of a ubiquitination pathway regulated by prolyl hydroxylase enzymes (PHDs). Translocation to the nucleus facilitates its binding to the hypoxia response element (HRE) and induces the expression of hypoxia related genes that encode e.g., vascular endothelial growth factor (VEGF) glucose transporters and glycolytic enzymes [7].

These HIF1 regulated genes trigger an endogenous neuroprotective response that has a role in neuronal survival [8]. It has been shown that stabilization of HIF1A through proteasome inhibition [9] or the use of heavy metals (cobalt or nickel) or metal chelation [8] attenuated hypoxia related damage. Furthermore, several studies concluded that oxyquinoline analogs are potent inhibitors of prolyl-4-hydroxylases and stabilize HIF1A [10,11,12].

Following the development of high-throughput phenotypic assays and subsequent gene expression analysis, the integration of cell-based assays with activity-based approaches allow us to identify small molecules interrogating cellular phenotypic alterations, changes in transcript signature or protein expression levels [13].

Previously, we developed a novel cell-microelectronic sensing technique (RT-CES)-based cytoprotective screening assay, and revealed Betti-bases as being a new subclass of potent cytoprotective agents; where 8-hydroxyquinoline (8HQ), benzocain, and benzaldehyde motifs emerged as suitable candidates for a hit-to-lead optimization [14]. Systematic preparative efforts led to the analogue Q50, which was proven to be highly potent in improving cardiac functional recovery of ischemic/reperfused myocardium in rats [15]. Recently, a 48-membered Betti library was synthesized to screen for neuroprotective compounds by the utilization of formic acid mediated industrial-compatible coupling with sets of aromatic primary amines such as anilines, oxazoles, pyridines, and pyrimidines, with (hetero)aromatic aldehydes and 8-hydroxiquinoline derivatives [16]. To the best of our knowledge, the asymmetric Betti reaction incorporating 8-hydroxyquinoline has not been studied so far.

Here, we describe the enantioselective synthesis of a potent 8-hydroxyquinoline-based Betti derivative, the cytoprotective effects of the enantiomers on neuronal cells as well as their effects on transcription and protein expression.

## 2. Results

### 2.1. Enantioselective Synthesis of Q134R and Q134S

**Scheme 1:**
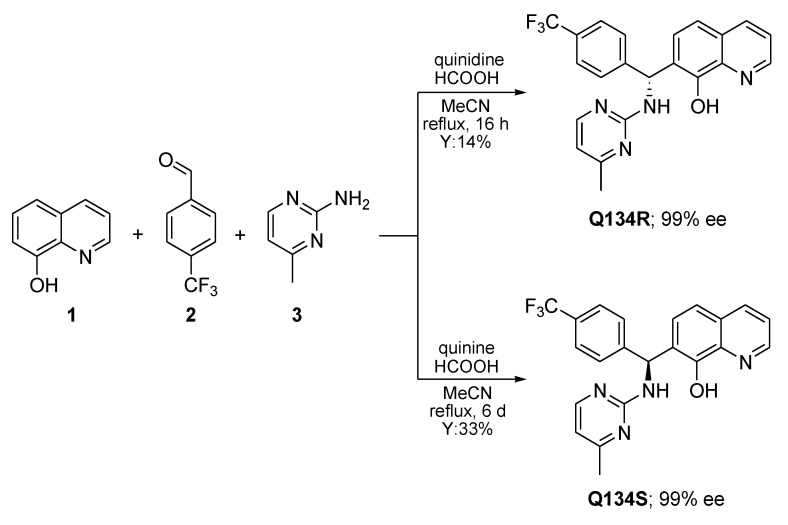
Enantioselective synthesis of Q134 enantiomers on 100 mmol scale.

As previously reported, a formic acid-mediated three-component assembly of 8HQ (**1**), 4-(trifluoromethyl)benzaldehyde (**2**), and 4-methylpyrimidin-2-amine (**3**) gave access to racemic Q134 [16]. In order to prepare pure enantiomers, cinchona alkaloids were firstly tested in the Betti-3CR, giving no conversion. However, excellent enantiomer ratios were obtained when quinidine or quinine were employed in the presence of formic acid (Scheme 1). It should be noted, that nearly a stoichiometric amount of catalyst and acid was applied, however, these reaction conditions were determined to be optimal for industrial synthesis, therefore further optimization was not performed. The chirality transfer might be explained via the formation of an ion pair between the catalyst and **1**, generating a chiral transition complex, which can react with the imine formed in situ. The absolute configuration of each enantiomer was supported by vibrational circular dichroism (VCD) and IR measurements. Moreover, the well-established protocol allowed the synthesis of Q134R and Q134S in an asymmetric fashion on a multigram scale [17].

### 2.2. Enantiomers of Q134 Show Similar Cytoprotective Activity

**Figure 1:**
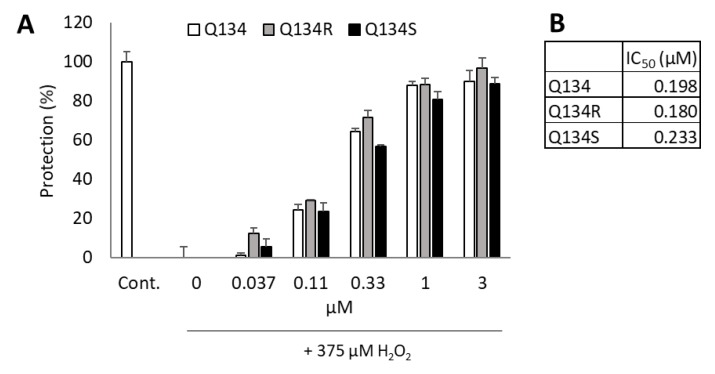
Cytoprotective activity of Q134 and its two enantiomers. (**A**) Cytoprotective activity 24 h after treatment. (**B**) Calculated IC_50_ values of the racemic mixture and the two enantiomers.

Q134R and Q134S, the two enantiomers of Q134 were tested in a real time cytoprotection assay. The two enantiomers and their racemic mixture showed comparable and potent activity in the assay with IC_50_ values of 180 nM and 233 nM, respectively. No enantiomer selectivity was determined in cytoprotective activity. Figure 1 shows cytoprotective activity 24 h after treatment.

### 2.3. Mitochondrial Membrane Depolarization Following Oxidative Stress Is Reversed by Treatment with Q134R and Q134S

**Figure 2:**
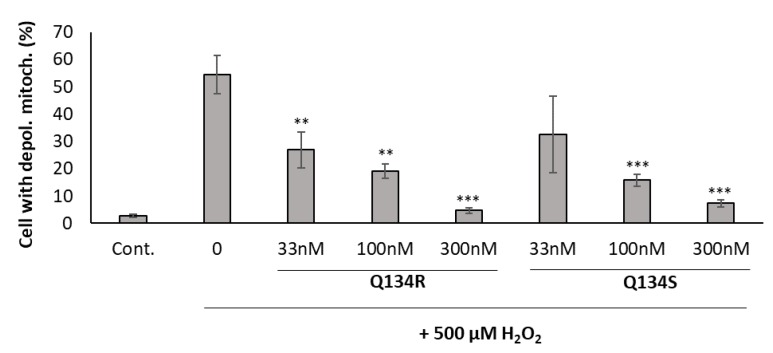
Mitochondrial membrane depolarization assay. Treatment with the synthesized enantiomers reversed the mitochondrial membrane potential changes caused by oxidative stress. Y axis represents the percentage of cells with depolarized mitochondria. ** *p* < 0.01; *** *p* < 0.001 (pairwise comparison of Q134R or Q134S treatment to only H_2_O_2_ treated cells; Student’s *t*-test).

Mitochondrial dysfunction is a prominent feature in neurodegenerative diseases, resulting in reactive oxygen species generation. Mitochondrial membrane potential (MMP) is affected by oxidative stress and may serve as a trigger for programmed cell death. Previously, we have shown that 8HQ analogs were able to normalize the mitochondrial membrane potential of U251 MG cells following oxidative stress in a dose dependent manner [16]. Here, we investigated whether Q134R and Q134S show any difference in their activity. U251 MG cells were treated with H_2_O_2_ and with an increasing dose of Q134R and Q134S and JC-1 staining was determined with flow cytometry after 2 h of incubation (Figure 2). Both compounds showed potent activity, but no difference was detected between Q134R and Q134S.

### 2.4. Q134R and Q134S Stabilize HIF1A Protein In Vitro

**Figure 3:**
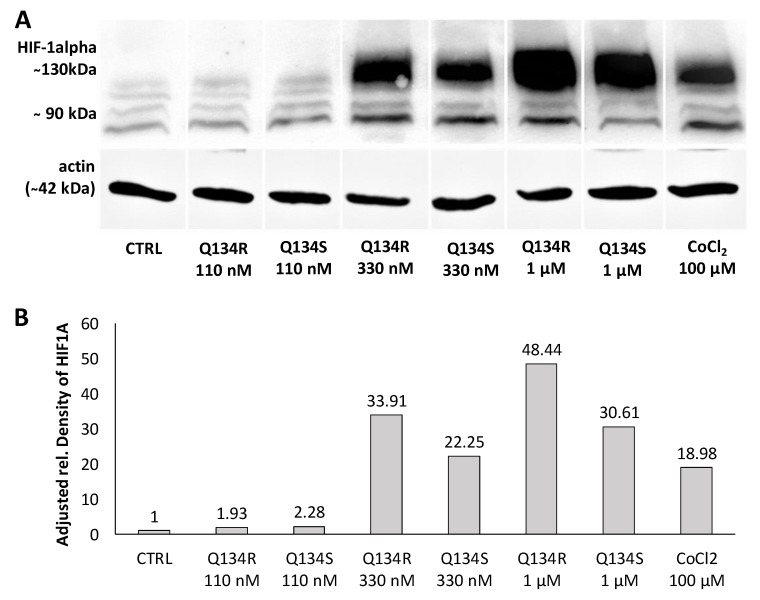
Q134R and Q134S treatment promotes the accumulation of hypoxia-inducible factor HIF1A protein. (**A**) Western blot analysis of treated U251 MG cells, (**B**) Quantification of HIF1A protein expression in treated U251 MG cells.

Our compounds showed potent activity in cytoprotection assays and previously we have shown that members of our original 8HQ library induced hypoxia related genes under HIF1A regulation. Previous studies showed that 8HQ analogs inhibit prolyl hydroxylases and as a result stabilize HIF1A protein. Therefore, we hypothesized that their activity was related to HF1 activity. U251 MG cells were treated with increasing doses of Q134R or Q134S, and with CoCl_2_ a compound known to stabilize HIF1A protein. After 3 h, cells were collected and HIF1A expression was determined by Western blot analysis. Robust bands were detected in case of both enantiomers, proving that Q134R and Q134S stabilized HIF1A in a dose dependent manner. As expected from the cytoprotection study, HIF1A protein stabilization showed no significant enantiomer selectivity (Figure 3).

### 2.5. Gene Expression Analysis. Q134R and Q134S Induce Hypoxia Related Genes and Glucose Transporter Expression

**Figure 4:**
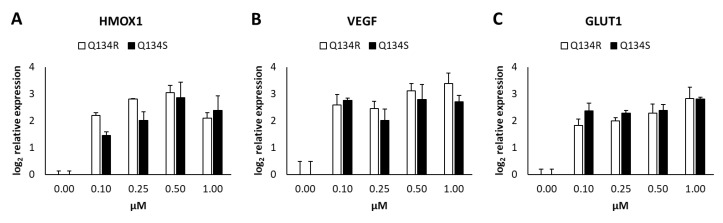
Quantitative real time PCR analysis of HIF1 regulated genes following Q134R and Q134S treatment in vitro. (**A**) HMOX1 gene expression, (**B**) VEGF gene expression, (**C**) GLUT1 gene expression.

We carried out a quantitative real-time PCR (QRT-PCR) study to determine any differences in modulation of gene expression levels of HIF1 regulated genes by the enantiomers of Q134. U251 MG cells were treated with an increasing dose of both Q134R or Q134S. After 6 h of incubation expression, levels of heme oxygenase 1 (HMOX1), vascular endothelial growth factor (VEGF), and glucose transporter 1 (GLUT1) were determined. Treatments with both enantiomers resulted in significant gene activation at concentrations, where cytoprotective activity was detected (see Figure 1). Again, no significant differences were detected in the activity of the two enantiomers (Figure 4).

### 2.6. Inhibition of HIF1A Translation Abolished Cytoprotective Activity

**Figure 5:**
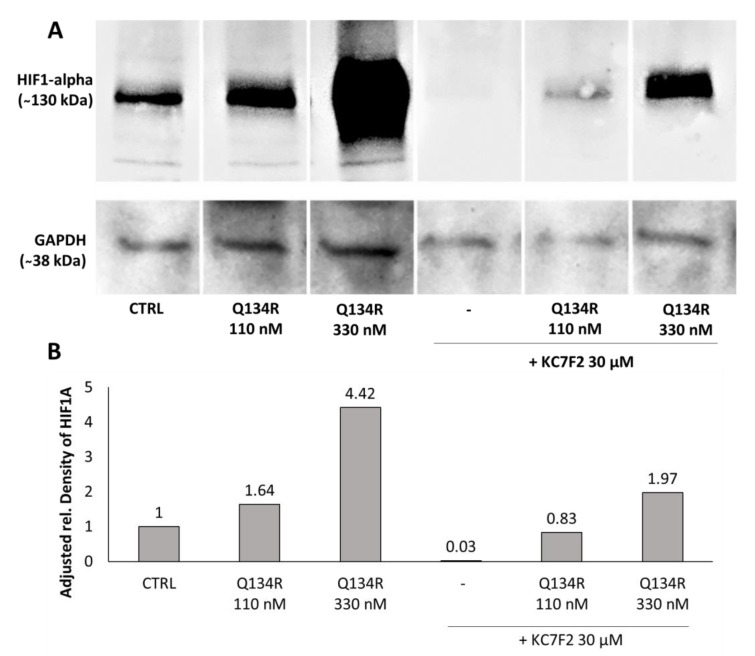
Inhibition of HIF1A translation; 24 h of KC7F2 treatment blocked HIF1A translation and depleted the treated cells from HIF1A. Q134R treatment only partially restored HIF1A expression. (**A**) Western blot analysis of treated U251 MG cells, (**B**) Quantification of HIF1A protein expression in treated U251 MG cells.

To validate HIF1A as a target of Q134 we designed an experiment where HIF1A translation was blocked and the cytoprotection activity of Q134R enantiomer was tested with no HIF1A present. We used KC7F2, a HIF1A translation inhibitor to deplete the cells from the HIF1A protein [18]. Western blot analysis showed that treatment with 30 µM of KC7F2 blocked HIF1A translation after 24 h (Figure 5, lane 4).

Consequently, we tested whether Q134R was able to restore HIF1A expression in cells depleted of HIF1A with KC7F2 (30 µM, 24 h). Western blot analysis showed (Figure 5) that Q134R was partially able to restore HIF1A, but only at the higher 330 nM concentration. The HIF1A level in cells treated with 110 nM Q134R was below control.

**Figure 6:**
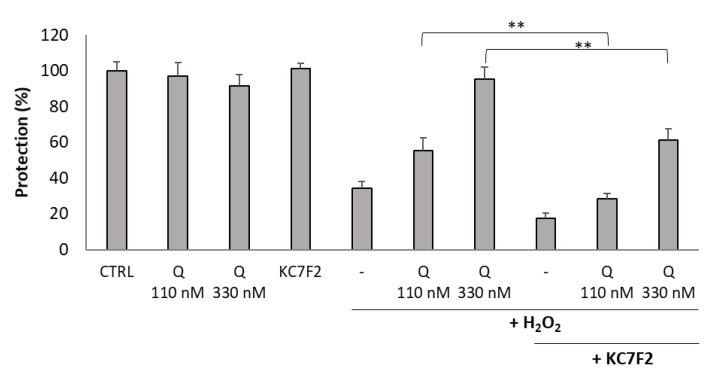
Inhibition of HIF1A translation abolished the cytoprotective activity of Q134R. U251 MG cells were pretreated with KC7F2 (30 µM, 24 h) to block HIF1A translation. Then, cells were treated with Q134R (Q 110 or 330 nM) and H_2_O_2_ (500 µM). ** *p* < 0.01 (Student’s *t*-test).

Finally, we tested how cytoprotection of Q134R was affected in cells depleted of HIF1A. Therefore, U251 MG cells were pretreated with KC7F2 for 24 h followed by treatment with Q134R and H_2_O_2_. An additional 24 h later cell viability was measured with the resazurin reagent. Similarly to the protein expression study (Figure 5), two concentrations were tested: 110 and 330 nM. Inhibitor treatment decreased Q134R activity at both tested concentrations. At 330 nM Q134R concentration, a 50% decrease of cytoprotective activity was detected while at 110 nM cytoprotection was abolished completely (Figure 6). For cytoprotection inhibition, similar results were recorded for Q134S (data not shown).

## 3. Discussion

Much importance in hypoxic signaling has been attributed to hypoxia-inducible factors, capable of activating a battery of genes including genes involved in glucose uptake and metabolism, extracellular pH control, angiogenesis, erythropoiesis, mitogenesis, and apoptosis [19]. Hypoxia-inducible factor-1 is a phosphorylation-dependent and redox-sensitive transcription factor that regulates neuroprotection during hypoxic conditions, and upregulation of HIF levels has been shown to be beneficial for ischemic diseases, stem cell proliferation [20], and transplantation [21]. As hypoxia is a prominent feature of neurodegenerative diseases such as AD, compounds that are able to modulate HIF-regulated targets may provide an avenue for the rescue of cells affected through their cytoprotective activity. Several research groups and drug discovery companies suggest that the HIF1 system is a potential therapeutic target to combat neurodegenerative diseases by designing and developing activators for cytoprotective protein induction.

We have selected Q134 as our candidate molecule from a previous study that included the development and investigation of a library of cytoprotective 8-hydroxyquinoline analogs. Here, we describe, for the first time, an asymmetric synthesis yielding enantiomer pure 8HQ products. The two synthesized enantiomers of Q134 were investigated for their cytoprotective activity and for their mechanism of action. It was apparent that both compounds acted identically as evidenced by their modulation of mitochondrial membrane potential, HIF1A protein and HIF1 regulated gene expression. Both Q134R and Q134S stabilized HIF1A at doses that were similar to their IC_50_ values in cytoprotection assays. This result corresponds well with previous findings that 8HQ analogs were inhibitors of PHDs that regulate HIF1A degradation [10,22]. As HIF1A stabilization was sufficient for cytoprotection in our assays (CoCl_2_ was cytoprotective, data not shown), we hypothesized that the HIF1 system was the main target that facilitated our compound’s cytoprotective activity. Depletion of cells from HIF1A using KC7F2, a HIF1A translation inhibitor resulted in complete loss of cytoprotective activity of Q134R. Further investigation of the direct target of Q134R may follow since our HIF1A protein expression experiments showed that KC7F2 completely blocked HIF1A production but subsequent Q134R treatment restored its presence partially. If Q134R only stabilized HIF1A, then no HIF1A should reappear after Q134R treatment in depleted cells. Either, KC7F2 is a partial inhibitor of translation and Q134R stabilizes the remaining HIF1A protein or Q134R interferes with the regulation of HIF1A translational inhibition of KC7F2.

Although we were not able to show significant differences in activity of the synthesized enantiomers, we selected Q134R as the clinical candidate, as it showed slightly better activity in cellular assays and in Western blot experiments.

It is crucial to obtain enantiomer pure versions of chiral molecules in drug development for their exact characterization in biological systems to identify target specific mechanism of action as well as to reveal their possible off-target effects. Therefore, development of an enantioselective synthesis is preferential against separation purification approaches. Moreover, this process provides an invaluable tool to produce these enantiomers in industrial scale.

Q134 is a central member of our central nervous system (CNS) drug development program that focuses on a multi-target approach to combat a complex disease such as AD. Therefore, to achieve an optimal co-modulation of several targets and to determine toxicity profiles it is important to evaluate the activity of each enantiomer independently. Detailed target identification and validation of the clinical candidate is in progress.

## 4. Materials and Methods

### 4.1. Enantioselective Synthesis of Q134R and Q134S

#### 4.1.1. General Information

NMR analysis and chromatography were performed as previously described [23]. Briefly, NMR spectra were recorded at 298 K on a Bruker Ascend 500 (Bruker, Billerica, MA, USA) with 5 mm BBO Prodigy Probe in CDCl_3_-*d*_1_ or DMSO-*d*_6_. The chemical shifts are reported in δ (ppm) relative to the internal standard tetramethylsilane (TMS) or the residual solvent signal. Data are reported as follows: chemical shift, multiplicity (s = singlet, d = doublet, t = triplet, q = quartet, m = multiplet, dd = doublet of doublet, dt = doublet of triplet, etc.) and integration. Chromatographic purification of the products was performed on Merck silica gel 60, particle size 0.063–0.200 mm. thin layer chromatography (TLC) was performed on fluorescent-indicating plates (aluminum sheets precoated with silica gel 60F254, 1.05554, Merck, (Kenilworth, NJ, USA), and visualization was achieved by UV light (254 nm) or by staining with basic potassium permanganate solution. Chiral HPLC measurements were performed by Shimadzu LC10 series (Shimadzu, Kyoto, Japan) using Phenomenex Lux 5u Cellulose-4 column (Phenomenex, Torrance, CA, USA). All reagents and solvents were commercially available and used without further purification.

#### 4.1.2. Synthesis of (R)-7-((4-methylpyrimidin-2-ylamino)(4-trifluoromethyl)phenyl)methyl)quinolin-8-ol (Q134R)

Quinidine (16.22 g, 50 mmol, 0.5 equiv.), formic acid (3.87 g, 84 mmol, 0.84 equiv.), 2-amino-4-methylpyrimidine (10.91 g, 100 mmol, 1.0 equiv.), 4-trifluoromethylbenzaldehyde (17.41 g, 100 mmol, 1.0 equiv.), and 8-hydroxyquinoline (17.42 g, 120 mmol, 1.2 equiv.) was added to a stirred solution of acetonitrile (180 mL) in a 500 mL round bottom flask. The reaction mixture was stirred at reflux temperature under an inert atmosphere for 16 h. The mixture was concentrated under reduced pressure to approx. one-third of the reaction mixture volume. The residue was diluted with dichloromethane (100 mL). The solution was extracted with 1 M NaOH solution (6 × 50 mL). The organic phase was evaporated. The residue was taken up in toluene (100 mL), and extracted with 3 M HCl solution (2 × 30 mL). Methyl tert-butyl ether (MTBE, 60 mL) was added to the combined aqueous layers was and the pH of the biphasic system was adjusted to 4 with 40% NaOH solution. The precipitated quinidine was filtered, and the filtrate layers were separated. The aqueous layer was washed with MTBE (2 × 20 mL), and the combined organic layers were dried over sodium sulfate, filtered, and evaporated. The residue was taken up in MeCN (30 mL), and the mixture was stirred at room temperature for 16 h. The precipitate was collected, yielding 7.61 g (18.5 mmol, HPLC purity: 95%) of racemic Q134. The filtrate was then evaporated, and the residue was triturated with isopropanol (40 mL) to give enantiomerically pure Q134R (5.67 g, 13.8 mmol, yield: 14%, HPLC purity: 98.9%, ee: 99%); ^1^H-NMR (500 MHz, DMSO-*d*_6_) δ 10.1 (1H, s), 8.85, (1H, dd), 8.30 (1H, dd), 8.15 (1H, d), 8.07 (1H, d) 7.75 (1H, d), 7.65 (2H, d), 7.60 (2H, d), 7.53 (1H, dd), 7.41 (1H, d), 7.09 (1H, d), 6.51 (1H, d), 2.25 (3H, s).

#### 4.1.3. Synthesis of (S)-7-((4-methylpyrimidin-2-ylamino)(4-trifluoromethyl)phenyl)methyl)quinolin-8-ol (Q134S)

Quinine (16.22 g, 50 mmol, 0.5 equiv.), formic acid (3.87 g, 84 mmol, 0.84 equiv.), 2-amino-4-methylpyrimidine (10.91 g, 100 mmol, 1.0 equiv.), 4-trifluoromethylbenzaldehyde (17.41 g, 100 mmol, 1.0 equiv.), and 8-hydroxyquinoline (17.42 g, 120 mmol, 1.2 equiv.) was added to a stirred solution of acetonitrile (180 mL) in a 500 mL round bottom flask. The reaction mixture was stirred at reflux temperature under an inert atmosphere for 6 days. The mixture was concentrated under reduced pressure to approx. one-third of the reaction mixture volume. The residue was diluted with dichloromethane (100 mL). The solution was extracted with 1 M NaOH solution (6 × 50 mL). The organic phase was evaporated. The residue was taken up in toluene (100 mL), and extracted with 3 M HCl solution (2 × 30 mL). MTBE (60 mL) was added to the combined aqueous layers and the pH of the biphasic system was adjusted to 4 with 40% NaOH solution. The precipitated quinidine was filtered, and the filtrate layers were separated. The aqueous layer was washed with MTBE (2 × 20 mL), and the combined organic layers were dried over sodium sulfate, filtered, and evaporated. The residue was taken up in MeCN (30 mL), and the mixture was stirred at room temperature for 16 h. The precipitate was collected, yielding 7.61 g (18.5 mmol, HPLC purity: 95%) of racemic Q134. The filtrate was then evaporated, and the residue was triturated with isopropanol (40 mL) to give Q134R (5.67 g, 13.8 mmol, yield: 14%, HPLC purity: 98.9%, ee: 99%); ^1^H-NMR (500 MHz, DMSO-*d*_6_) δ 10.1 (1H, s), 8.85, (1H, dd), 8.30 (1H, dd), 8.15 (1H, d), 8.07 (1H, d) 7.75 (1H, d), 7.65 (2H, d), 7.60 (2H, d), 7.53 (1H, dd), 7.41 (1H, d), 7.09 (1H, d), 6.51 (1H, d), 2.25 (3H, s).

### 4.2. Cell Culture

U251 MG human glioblastoma cell line was a gift from Szabolcs Bellyei, University of Pecs (originally obtained from American Type Culture Collection, Manassas, VA, USA). Cells were grown at 37 °C under 5% of CO_2_ and 100% humidity in RPMI medium supplemented with 10% fetal calf serum (FCS) (Sigma-Aldrich, Budapest, Hungary), and penicillin-streptomycin antibiotics (Life Technologies, Carlsbad, CA, USA) [24].

### 4.3. Real-Time CellE Sensing (RT-CES) Cytoprotection Assay

RT cytoprotection assay was performed as previously described [14,16,25]. Briefly, RT-CES 96-well E-plate (BioTech Hungary, Budapest, Hungary) was coated with gelatin solution (0.2% in PBS, phosphate buffer saline) for 20 min at 37 °C, then gelatin was washed twice with PBS solution. Growth media (50 μL) was then gently dispensed into each well of the 96-well E-plate for background readings by the RT-CES system prior to the addition of 50 μL of the cell suspension containing 10^4^ U251 MG cells. Plates were kept at room temperature in a tissue culture hood for 30 min prior to insertion into the RT-CES device in the incubator to allow cells to settle. Cell growth was monitored overnight by measurements of electrical impedance every 15 min. Continuous recording of impedance in cells was reflected by cell index value. The next day cells were co-treated with 375 μM H_2_O_2_ and test compounds. Treated and control wells were dynamically monitored over 48 h by measurements of electrical impedance every 5 min. The raw plate reads for each titration point were normalized relative to the cell index status right before treatment. Each treatment was repeated in 3 wells per plate during the experiments. Protection values were calculated with relation to normalized cell index values of non-treated (100%) and hydrogen peroxide treated cells (0%). The presented IC_50_ values (half maximal inhibitory concentration) were determined based on dose-response curves plotted in GraphPad Prism^®^ 5.

### 4.4. Detection of Mitochondrial Membrane Potential

Mitochondrial membrane potential was measured as described previously [16,26]. U251 cells (6 × 10^4^) were plated in 24-well tissue culture plates (Corning Life Sciences, Tewksbury, MA, USA) in RPMI 10% FCS and were treated in 500 µL media containing 500 µM H_2_O_2_ with or without test compounds. Untreated controls cells were supplemented with 500 µL cell culture media. After 2 h, the supernatants were harvested. Cells were washed with PBS, trypsinized, pooled with the corresponding supernatant, and centrifuged (2000 rpm, 5 min). Pellet was resuspended and incubated for 15 min in 5 µg/mL JC-1 (5,5’,6,6’-tetrachloro-1,1’,3,3’-tetraethylbenzimidazolocarbocyanine iodide, Chemodex) containing media in a final volume of 300 µL at 37 °C. Finally, using FL2 (585/42 nm)–FL1 (530/30 nm) channels, the red–green fluorescence intensity of 6 × 10^3^ events was acquired immediately on FACSCalibur (Becton Dickinson, Franklin Lakes, NJ, USA) flow cytometer. Data were analyzed using CellQuest Pro software (version 6.0, Becton Dickinson) gating out debris. Bar graphs show the percentage of FL1 positive cells.

### 4.5. Gene Expression Analysis

Gene expression measurements were performed as previously described [16]. Briefly, U251 MG cells were seeded in 24-well microtiter plates with 10^5^ cells/well density and incubated overnight. Cells were treated with either vehicle (solvent control; ≤0.01% DMSO) or with Q134 enantiomers: Q134R and Q134S at 0.1, 0.25, 0.5, and 1 µM concentration. After 6 h of incubation the medium was removed and cells were rinsed with PBS. Cells were collected for RNA isolation in RA1 Lysis Buffer (Macherey-Nagel, Düren, Germany) supplemented with 1% beta-mercaptoethanol. Total RNA was purified with the Direct-zol kit (Zymo Research, Irvine, CA, USA) according to the manufacturer’s protocol. Total RNA was converted into cDNA using the High-Capacity cDNA Archive Kit (Applied Biosystems, Foster City, CA, USA) in a volume of 10 µL. Then 0.75 µL cDNA template was used (19 ng) in each PCR reaction. Quantitative real-time PCR was performed on the Light Cycler 96 instrument (Roche) with gene-specific primers and SybrGreen protocol. NormFinder software [27] was used to calculate the most suitable housekeeping genes (TUBB and PPIA) for relative expression ratio calculation. Table 1 lists the used primer sequences.

### 4.6. Western Blot Analysis

Western blot analysis was performed as described previously [28]. Briefly, U251 MG cells were treated with test compounds or inhibitor (KC7F2, Selleckchem, Houston, TX, USA). Western blot samples were obtained by scraping 10^6^ cells from 6-well plates in 250 µL of hot 1× Laemmli buffer. Then 20 µL samples were run on a 4–15% SDS-PAGE and blotted onto Immobilon P PVDF (polyvinylidene fluoride) membrane (0.45 µm pore size, Millipore, (Budapest, Hungary). After blocking with 0.5% nonfat dry milk powder in PBS containing 0.1 % Tween 20, membranes were labeled 1:700 with HF1 alpha antibody (PA1-16601, ThermoFisher, (Waltham, MA, USA). As secondary antibodies goat anti-rabbit whole IgG antibodies were used (111-035-003, Jackson ImmunoResearch, (Cambridgeshire, UK)

Proteins were visualized using the ECL system (Luminata Forte Western HRP substrate, Millipore, Budapest, Hungary, # WBLUF0100).

Quantification was carried out using ImageJ software by referring the relative density of HF1 alpha bands to the untreated sample relative density. Data are shown as adjusted relative density by referring the relative intensity of HF1 alpha bands to the corresponding loading control intensities (Actin: Sigma-Aldrich, A2103 or GAPDH: Invitrogen, PA1-987).

### 4.7. Resazurin Viability Assay

Endpoint viability assays were performed as described previously [16]. For cytoprotection assays, 10^4^ U251 MG cells were seeded into each well of 96-well cell culture plates (Costar, Corning Inc., Tewksbury, MA, USA) in culture medium containing 10% FCS. The following day cells were treated with test compounds in the presence of 500 μM H_2_O_2_ (Sigma-Aldrich). Cell viability was recorded 24 h after treatment. Resazurin reagent (Sigma-Aldrich) was dissolved in phosphate buffered saline (PBS, pH 7.4) at 0.15 mg/mL concentration, sterile filtered (0.22 µm, Merck Millipore), aliquoted, and stored at −20 °C. Samples were treated with a final concentration of 25 µg/mL resazurin. After 2 h of incubation at 37 °C 5% CO_2_, fluorescence (530 nm excitation/580 nm emission) was recorded on a multimode microplate reader (Cytofluor4000, PerSeptive Biosytems, Framingham, MA, USA). Viability was calculated in relation to untreated control cells and blank wells containing media without cells.

### 4.8. Statistical Analysis

Statistical analysis was performed using two-tailed, Student’s *t*-test to evaluate the statistical significance (set at * *p* < 0.05, ** *p* < 0.01, *** *p* < 0.001) between two given experimental groups.

## 5. Conclusions

We selected a candidate molecule from our library of cytoprotective 8-hydroxyquinoline analogs. We developed an asymmetric synthesis methodology to produce enantiomer pure 8HQ products for the first time. The biological activity of the two synthesized enantiomers of Q134 were investigated and found to be identical in relation to the HIF1 system. We have shown that HIF1A was a necessary component for the cytoprotective activity of Q134R and that its activity is based on the stabilization of HIF1A protein.

## 6. Patents

Compounds described in the current study are protected by patent #WO2011148208 and WO2016162706 A1.

## Figures and Tables

**Table 1:** Primer sequences for quantitative real-time (QRT)-PCR analysis.

Gene Name	Abbreviation	Forward_Sequence	Reverse_Sequence
Tubulin beta class I	TUBB	ataccttgaggcgagcaaaa	ctgatcacctcccagaacttg
Peptidylprolyl isomerase A	PPIA	atgctggacccaacacaaat	tctttcactttgccaaacacc
Heme oxygenase 1	HMOX1	ggcagagggtgatagaagagg	agctcctgcaactcctcaaa
Vascular endothelial growth factor	VEGF	gcagcttgagttaaacgaacg	ggttcccgaaaccctgag
Solute carrier family 2 member 1	GLUT1	ccccatcccatggttcatc	tgaggtccagttggagaagc
